# Prisons as Hotspots of Tuberculosis: A Silent Threat in Ecuador

**DOI:** 10.3390/pathogens15101029

**Published:** 2026-09-30

**Authors:** Carolina Viteri-Dávila, Deyaneira Cevallos-Arteaga, Lennys Berutti-Suárez, Carlos Bastidas-Caldes

**Affiliations:** 1 Clinical Laboratory Career, Universidad Politécnica Estatal del Carchi, Tulcán 040101, Ecuador; deyaneira.cevallos@upec.edu.ec (D.C.-A.); lennys.berutti@upec.edu.ec (L.B.-S.); 2 School of Medicine, Universidad Espíritu Santo, Samborondón 092301, Ecuador

**Keywords:** prisons, tuberculosis, Ecuador, drug resistance, MDR-TB, overcrowding, molecular epidemiology, incarceration

## Abstract

Tuberculosis (TB) has become established as a critical public health crisis within Ecuador’s penitentiary system, with incidence and prevalence rates that drastically exceed those of the general population. This narrative review aims to synthesize the available evidence regarding the epidemiological burden, structural determinants, and transmission dynamics of TB in Ecuadorian prisons. We conducted a structured literature search in PubMed, Scopus, and Web of Science, using keywords related to tuberculosis, prisons, and Ecuador. Within the literature review, a severe epidemiological burden was found when evaluating 2015–2016 TB control and treatment risks at CPL Guayas No. 1 (Ecuador’s largest prison), which reported an incidence of 3947 per 100,000 inmates, approximately 68 times the national average. Structural determinants, especially extreme overcrowding exceeding 140% in some facilities, poor ventilation, and limited access to rapid diagnostics such as Xpert MTB/RIF, enable prisons to act as amplifiers of pathogen transmission. Furthermore, the review highlights the circulation of rifampicin-resistant strains and the predominance of lineage 4 (LAM sublineage), which is strongly associated with drug resistance in the Ecuadorian context. Because the infection transcends prison walls through released inmates, staff, and visitors, the disease spreads into the general community, increasing state costs and control challenges. In conclusion, evidence demonstrates that structural deficiencies and extreme overcrowding drive this health crisis. Based on these findings, urgent structural interventions are recommended to reduce overcrowding and strengthen healthcare infrastructure, integrating criminal justice policies with public health strategies.

## 1. Introduction

Tuberculosis (TB) remains one of the top ten causes of mortality worldwide. The World Health Organization (WHO) estimated that in 2023 there were 10.8 million new cases of tuberculosis (TB) globally [1]. More than 95% of TB-related deaths occur in low- and middle-income countries, where intensive efforts to provide access to quality healthcare remain insufficient, regardless of social environment, age, sex, or ability to pay [2]. In the Americas, TB caused approximately 342,000 new cases and 25,000 deaths in the same year [3].

TB remains one of the most prevalent and severe infections in immunocompromised populations, particularly in people living with HIV [2]. In the region, 218,700 TB cases were reported in 2015, of which approximately 9.49% (21,885 cases) corresponded to TB/HIV coinfection [2]. More recent official national TB/HIV coinfection data are not available; therefore, the 2015 figure remains the most current estimate reported by national authorities. The overall landscape of global TB control is further complicated by the high prevalence of HIV infection and the emergence of multidrug-resistant (MDR) or extensively drug-resistant (XDR) forms in all regions, highlighting the need for national TB programs tailored to the specific needs of high-risk groups [2].

Ecuador has a medium TB burden. In 2023, WHO estimated TB incidence at 58 cases and a mortality rate of 4.7 deaths per 100,000 inhabitants [1]. This persistent epidemiological burden represents a significant challenge to progress toward WHO elimination goals. However, the country lacks precise information on annual TB cases among people deprived of liberty [3,4]; discrepancies between peer-reviewed findings and official census-based data indicate surveillance gaps rather than methodological errors.

People deprived of liberty (PDL) are a highly vulnerable risk group, as they face an extremely high TB burden, with incidence rates up to 27 times higher than those of the general population in South America [4]. In Ecuador, this critical trend is reflected in the prison system, where TB is recognized as a severe public health crisis [5]. The prevalence of TB in Ecuadorian prisons has shown wide ranges, between 1.4% and 4.7%, figures that exceed by several tens the rates observed in the non-incarcerated population [6]. Epidemiological evidence underscores that prison environments function as high-risk transmission settings due to conditions such as overcrowding, errors in the direct care of PDL, delays in diagnosis, and limited access to healthcare services, among others. These factors facilitate the progression from infection to active disease and its subsequent transmission [7]. These are some of the reasons why TB treatment is often unsuccessful in this affected group; consequently, this leads to the spread of the disease within the community and increased treatment costs for the State [6,7].

In Latin America, the incarcerated population has nearly quadrupled since 1990, with a 206% increase in Central and South America between 2000 and 2018, which has progressively concentrated TB in this population [8]. Notifications of TB cases among PDL have increased by more than 250% since 2000 in the region, with a growing proportion of all reported cases occurring in prisons—rising from 6.6% in 2014 to 9.4% in 2019 [8]. The total impact of incarceration is underestimated when focusing solely on cases reported within prisons, as infection spreads to the community once individuals are released, or through prison staff and visitors [9].

The Epidemiological Prevention and Control Strategy for TB aims to ensure detection, diagnosis, and treatment of the disease to reduce morbidity and mortality and prevent the emergence of drug resistance. In Ecuador, the National Directorate of Epidemiological Surveillance supports this process through case control and monitoring [10,11].

According to Ecuador’s Ministry of Public Health, 1115 TB cases were detected in penitentiary centers nationwide. The provinces with the highest incidence of TB are Guayas, El Oro, and Los Ríos, which account for all reported cases of drug-susceptible tuberculosis. This may be due to the presence of vulnerable populations in these provinces, such as individuals with health conditions like human immunodeficiency virus (HIV) infection, people with diabetes, limited economic resources, and other risk factors [10,12].

Despite increasing recognition of tuberculosis as a major public health problem in prison populations, comprehensive syntheses of the available evidence in Ecuador remain limited. Most existing studies focus on specific prison facilities or individual epidemiological indicators, making it difficult to obtain an integrated perspective of the structural and epidemiological factors driving tuberculosis transmission in incarcerated populations.

This narrative review aims to synthesize available evidence on the epidemiology, transmission dynamics, and structural determinants of tuberculosis in Ecuadorian prison settings and discuss their implications for tuberculosis control and public health policy.

## 2. Methodological Approach for Literature Search

This narrative review synthesizes available scientific evidence on the epidemiology of tuberculosis in prison populations, with a specific focus on Ecuador and the broader context of Latin America. Although this is a narrative synthesis, we applied a structured search strategy with explicit keywords, databases, and eligibility criteria (detailed below) to identify relevant literature, without following a fully quantified systematic screening process.

### 2.1. Search Strategy and Data Sources

We conducted a structured literature search in the electronic databases PubMed, Scopus, and Web of Science, using Google Scholar to capture grey literature and complement the primary databases. We conducted the final database search in May 2026. The exact search string utilized across databases was adapted for each interface but fundamentally relied on the following Boolean structure: (“tuberculosis” OR “TB” OR “multidrug-resistant tuberculosis”) AND (“prison” OR “incarcerated” OR “penitentiary”) AND (“Ecuador” OR “Latin America” OR “HIV coinfection”).

### 2.2. Eligibility Criteria

To ensure the relevance and quality of the synthesized data, we established specific inclusion and exclusion criteria.

### 2.3. Inclusion Criteria

Peer-reviewed original research, epidemiological studies, systematic reviews and meta-analyses, and official government or public health reports (particularly from Ecuadorian health authorities); studies published between January 2000 and May 2026; articles published in English or Spanish; and studies directly addressing tuberculosis transmission, incidence, prevalence, molecular epidemiology, and risk factors in prison populations were included.

### 2.4. Exclusion Criteria

Editorials, opinion pieces, and unverified preprints; studies with inaccessible full texts; and research outside the epidemiological scope of the review were excluded.

### 2.5. Study Selection and Data Extraction

We initially screened titles and abstracts for relevance, then evaluated eligible articles in full text. We identified and removed duplicates across databases using the reference management software Zotero 10.0.03.

Because this work was conceived as a narrative rather than a systematic review, we did not generate a formal PRISMA-type flow diagram quantifying records identified, screened, and excluded at each stage. Consistent with this narrative design, the search strategy aimed to identify the most relevant and representative literature on the topic rather than to produce an exhaustive or fully reproducible enumeration of all available records. A total of 18 sources meeting the eligibility criteria were ultimately selected and are cited throughout this review.

This review draws on secondary analyses of published studies, national epidemiological databases, and meta-analyses, as reported in the referenced literature [2]. We used a secondary data synthesis approach to extract and compare prevalence and incidence rates, risk factors, patterns of *Mycobacterium tuberculosis* genotypes, disease outcomes, and the potential impact of prison policies on transmission dynamics [6]. To provide local epidemiological context within the Ecuadorian prison system, we incorporated evidence from a cross-sectional study conducted in 2019 at the Guayaquil penitentiary center, the prison with the highest population density in the country. In that study, researchers evaluated 634 inmates entering the facility between February and April to estimate prevalence, incidence, and risk factors associated with pulmonary tuberculosis [6]. We used odds ratios and logistic regression analyses to identify predictive factors associated with active disease.

We obtained these isolates from the general Ecuadorian population through the national reference laboratory, not specifically from incarcerated individuals. We included these data not to draw direct intra-prison epidemiological conclusions, but to establish the national baseline of circulating strains. Understanding the national molecular epidemiology provides critical context regarding the lineages that enter the penitentiary system from the community and subsequently amplify in overcrowded conditions.

Regional and global evidence on the relationship between incarceration and tuberculosis burden was incorporated from systematic reviews and mathematical modeling studies estimating the incidence and prevalence of tuberculosis in prison populations worldwide and in Latin America [4,8]. These models included counterfactual scenario analyses that estimate the transmission population attributable fraction associated with incarceration and the potential impact of alternative public health policies.

Because this work was a narrative synthesis rather than a systematic review, we did not conduct a formal risk-of-bias assessment or quantitative meta-analysis. Instead, the objective was to integrate the most relevant epidemiological and conceptual evidence to contextualize tuberculosis transmission dynamics in prison populations.

## 3. Results

### 3.1. Structural Conditions of Ecuadorian Prisons

Ecuador’s prison system has experienced rapid population growth in recent years, generating substantial levels of overcrowding across correctional facilities. According to official statistics from the National Service for the Comprehensive Care of Adults Deprived of Liberty (SNAI) [13] and the Inter-American Commission on Human Rights (IACHR) [14], the national prison population increased from 32,941 individuals in 2024 to 35,327 in 2025, exceeding the system’s operational capacity. During this period, prison capacity was estimated at 27,714 in 2024 and 27,138 in 2025, bringing national overcrowding levels close to 30%.

Independent human rights monitoring has documented even more severe conditions at the facility level. The Inter-American Commission on Human Rights reported overcrowding rates exceeding 100% in several Ecuadorian prisons, including 141% in Los Ríos prison and 124.6% in Guayas No.5 prison. Such structural conditions substantially increase the risk of airborne disease transmission, particularly tuberculosis, due to prolonged confinement in poorly ventilated spaces and limited access to adequate health services.

### 3.2. Tuberculosis Burden in Ecuadorian Prisons

Tuberculosis represents one of the most significant infectious diseases affecting incarcerated populations in Ecuador. Epidemiological studies conducted in the country’s largest penitentiary complex (CPL Guayas No.1) have reported extremely high tuberculosis incidence rates compared with the general population. Although these extreme rates from CPL Guayas No.1 cannot be uniformly extrapolated to all Ecuadorian prisons, they highlight the severe epidemiological vulnerabilities in the most overcrowded facilities in the national system. Furthermore, regarding drug-resistant tuberculosis, there is a critical lack of prison-specific drug-resistance prevalence data in Ecuador, highlighting an urgent need for targeted molecular surveillance within these facilities.

Table 1 summarizes the key structural and epidemiological indicators associated with tuberculosis risk in Ecuadorian prisons.

Available epidemiological evidence indicates that tuberculosis incidence in Ecuadorian prisons can reach 3947 cases per 100,000 incarcerated persons [7], approximately 68 times higher than the national incidence reported in the general population [1]. While this figure originates from a specific facility-level study conducted at CPL Guayas No. 1, it illustrates the disproportionate burden of tuberculosis experienced by incarcerated populations nationally and underscores the role of correctional facilities as important epidemiological hotspots for disease transmission, particularly under the extreme overcrowding conditions documented across the prison system [13,14].

### 3.3. Tuberculosis Transmission Dynamics in Prison Settings

Several studies have identified structural and epidemiological factors that sustain tuberculosis transmission within prison environments, including overcrowding, poor ventilation, delayed diagnosis, and limited capacity to isolate infectious individuals. Evidence from prison-based epidemiological investigations indicates that tuberculosis incidence frequently increases during incarceration rather than solely reflecting infections acquired prior to prison entry. This suggests that prisons may function not only as high-risk transmission settings but also as amplifiers of transmission dynamics.

Limited diagnostic capacity further exacerbates this situation. Access to rapid molecular diagnostic tests such as Xpert MTB/RIF remains inconsistent across prison facilities, and delays between diagnosis and treatment initiation may extend from days to several months in some cases. These gaps in diagnostic and treatment capacity contribute to prolonged periods of infectiousness and facilitate the propagation of tuberculosis within prison populations.

### 3.4. Drug-Resistant Tuberculosis in Incarcerated Populations

Drug-resistant tuberculosis represents an additional challenge for tuberculosis control in prison settings. Molecular epidemiological studies conducted in Ecuador have reported the circulation of rifampicin-resistant strains of *Mycobacterium tuberculosis* among incarcerated populations.

Genotyping analyses of clinical isolates collected in Ecuador have identified the predominance of lineage 4 and sublineage LAM among circulating strains. These findings highlight the importance of routine drug susceptibility testing and molecular surveillance to monitor transmission patterns and detect emerging resistant strains.

The combination of high transmission rates, delayed diagnosis, treatment interruptions, and overcrowded prison environments creates conditions that may facilitate the emergence and dissemination of drug-resistant tuberculosis within correctional systems. Consequently, prisons represent critical intervention points for tuberculosis control strategies at both national and regional levels.

Recent molecular epidemiology studies provide additional insights into the genetic diversity of *M. tuberculosis* circulating in Ecuador. A nationwide analysis of 373 clinical isolates from the general Ecuadorian population (not the prison system; see Methods) showed that lineage 4 predominates overwhelmingly in the country, accounting for about 98% of strains, with the LAM and Haarlem sublineages representing most circulating genotypes [15]. The LAM sublineage alone accounted for nearly half of the isolates and showed the strongest association with anti-tuberculosis drug resistance, suggesting that specific genetic backgrounds may contribute to the emergence and persistence of drug-resistant tuberculosis in the Ecuadorian context.

## 4. Discussion

Tuberculosis in prison populations represents a significant and frequently underestimated contributor to the regional tuberculosis burden. The findings synthesized in this review highlight that the exceptionally high burden of tuberculosis observed in Ecuadorian prisons is not an isolated national phenomenon but part of a broader regional pattern across Latin America. Mathematical modeling studies have demonstrated that the expansion of incarceration policies since the 1990s has generated a substantial and previously underrecognized tuberculosis burden in the region [8]. In 2019 alone, an estimated 30,241 excess incident tuberculosis cases were attributed to rising incarceration rates across six Latin American countries, representing nearly a quarter of the regional tuberculosis transmission burden [8].

The results indicate that Ecuador follows the same epidemiological trajectory. Tuberculosis incidence rates in prison settings exceed those observed in the general population by several orders of magnitude, confirming that correctional facilities act as institutional amplifiers of transmission [5,16]. Evidence from Guayaquil’s largest penitentiary complex shows that while tuberculosis prevalence at prison entry is already elevated, incidence increases markedly during incarceration, demonstrating that prisons are not merely passive settings receiving infected individuals but environments where transmission chains are actively sustained [6].

Structural conditions within the Ecuadorian prison system play a central role in shaping this epidemiological scenario. Official statistics from the National Service for the Comprehensive Care of Adults Deprived of Liberty (SNAI) indicate that the prison population increased from 32,941 individuals in 2024 to 35,327 in 2025, exceeding the prison system’s operational capacity and resulting in overcrowding levels approaching 30% nationwide. These conditions substantially increase the risk of airborne disease transmission due to prolonged confinement in poorly ventilated spaces and limited access to adequate healthcare services.

Independent human rights monitoring has documented even more severe conditions at the facility level. Reports from the Inter-American Commission on Human Rights have identified extreme overcrowding in several Ecuadorian prisons, including occupancy rates exceeding 140% in some facilities. These structural conditions have been associated with severe limitations in access to healthcare, delayed diagnosis of infectious diseases, and interruptions in treatment continuity, factors that collectively create environments highly conducive to tuberculosis transmission within correctional facilities.

Figure 1 summarizes the interaction between structural determinants, transmission mechanisms within prisons, and spillover into surrounding communities. Overcrowding, limited ventilation, delayed diagnosis, and insufficient healthcare infrastructure facilitate prolonged infectious periods and sustained transmission among incarcerated individuals. These structural conditions reflect broader patterns of social and institutional vulnerability frequently documented in correctional environments [17].

Another critical factor amplifying tuberculosis transmission in prison settings is the presence of multiple individual risk factors within incarcerated populations. HIV coinfection has been identified as the most significant predictor of active tuberculosis in Ecuadorian prisons, with extremely high associated risks [6].

Additionally, as well-established in the broader international prison literature [1,4], high levels of alcohol and tobacco consumption among inmates increase immunological vulnerability and the probability of progression from latent infection to active disease. Similar epidemiological patterns have been documented in other prison systems worldwide, where tuberculosis incidence in incarcerated populations may exceed that of the general population by more than twenty-fold.

The emergence and circulation of drug-resistant tuberculosis further complicates disease control in correctional settings. Molecular epidemiological studies conducted in Ecuador have identified a predominance of *Mycobacterium tuberculosis* lineage 4, particularly the LAM sublineage, among circulating strains [13]. The concentration of these lineages in prison populations suggests that correctional facilities may function as focal points for the amplification and dissemination of resistant strains, posing additional challenges for national tuberculosis control strategies.

Importantly, the consequences of tuberculosis transmission within prisons extend far beyond correctional facilities themselves. Individuals who have experienced incarceration maintain elevated tuberculosis risk for several years after release, contributing to sustained transmission within surrounding communities. This dynamic reinforces the concept that prison health is inseparable from community health and that tuberculosis control strategies that ignore prison populations are inherently incomplete.

Evidence from genomic epidemiology further supports the role of prisons as key drivers of tuberculosis transmission in Latin America. A large population-based genomic study conducted in Brazil demonstrated extensive transmission links between incarcerated individuals and surrounding communities, revealing that tuberculosis strains circulating in prisons frequently contribute to broader community transmission networks.

Importantly, the study showed that incarceration acts not only as a setting for transmission but also as a structural amplifier of regional tuberculosis epidemics. These findings highlight how correctional facilities can sustain transmission chains that extend beyond prison walls and reinforce the concept that effective tuberculosis control strategies must incorporate prison populations as central components of national control programs [16].

Current control strategies in Ecuador remain insufficient to address the structural determinants driving tuberculosis transmission in prison environments. There is direct evidence that specific biomedical interventions, such as systematic active case finding (ACF) combined with the use of rapid molecular diagnostics, are highly effective in reducing diagnostic delays and improving case detection in prison settings; in fact, the addition of molecular techniques like GeneXpert into Ecuadorian diagnostic algorithms has improved resource optimization and enabled the early diagnosis of MDR-TB [18]. This is particularly relevant considering that prisoners are recognized among the highly vulnerable population groups heavily affected by the TB burden [16]. Although interventions such as mass screening at prison entry and during incarceration can improve case detection, evidence suggests that these measures alone are insufficient to interrupt transmission cycles. Structural interventions addressing overcrowding, prison infrastructure, and healthcare capacity are therefore essential.

### International Experience in Prison-Based Tuberculosis Screening and Its Applicability to Ecuador

International experience offers concrete, quantifiable evidence on which screening strategies could realistically be adapted to the Ecuadorian penitentiary system. In Peru, the National Tuberculosis Program launched a nationwide active case-finding (ACF) initiative across 18 correctional facilities between January and September 2024, screening more than 38,000 incarcerated individuals using chest radiography combined with rapid molecular diagnostic tests [19]. This program identified an overall TB prevalence of 2.8% among screened participants, and notably, 40.9% of detected cases occurred in individuals with abnormal radiographic findings but no reported symptoms, demonstrating that purely symptom-based screening—the approach still predominant in many Latin American prison systems, including Ecuador—systematically misses a substantial proportion of active cases [19]. The program also identified clusters of rifampin-resistant tuberculosis in specific facilities, reinforcing the need for molecular testing rather than smear microscopy alone as the diagnostic backbone of any screening strategy [19].

A complementary mathematical modeling study comparing systematic screening strategies across prisons in Brazil, Colombia, and Peru provides direct guidance on which approach maximizes impact relative to cost [20]. The model demonstrated that biannual screening combined with entry and exit screening, using chest X-ray with computer-aided detection (CXR-CAD), could reduce tuberculosis incidence within prisons by 61–87% and at the broader population level by 18–28% [20]. Critically, the incremental cost per disability-adjusted life year (DALY) averted was substantially lower in Peru than in Brazil or Colombia, and in settings lacking CXR-CAD infrastructure, pooled sputum testing with GeneXpert emerged as a cost-effective alternative strategy [20]. These findings are directly relevant to Ecuador, where the Peruvian resource profile—rather than the higher-infrastructure Brazilian model—represents the more realistic benchmark for feasibility.

However, these strategies’ direct transferability to Ecuador faces specific structural barriers that must be acknowledged. First, Peru’s program operated as a centrally coordinated national protocol; Ecuador currently lacks an equivalent standardized, nationwide ACF program and instead relies on opportunistic, facility-dependent use of GeneXpert [18]. Second, the overcrowding levels documented in Ecuadorian facilities—reaching 141% in Los Ríos and 124.6% in Guayas No. 5 (Table 1)—exceed typical occupancy levels assumed in the Peruvian and Brazilian modeling scenarios, which may reduce the isolation and containment benefits normally associated with early case detection, since screened individuals often cannot be physically separated pending treatment initiation. Third, the Peruvian program depended on radiography equipment and trained personnel deployed across 18 facilities simultaneously; Ecuador’s prison health infrastructure does not currently have comparable diagnostic imaging capacity distributed at that scale, meaning that a lower-cost, symptom-plus-GeneXpert model (rather than the CXR-CAD-based approach) may be the more immediately implementable entry point.

Currently, tuberculosis control in Ecuadorian prisons relies primarily on passive case detection—that is, testing individuals only after they present with clinical symptoms—supplemented by inconsistent, facility-dependent access to GeneXpert [16]. This falls considerably short of the systematic, protocolized, and centrally coordinated ACF model demonstrated in Peru [19], and it explains why the 40.9% of asymptomatic-but-radiographically-positive cases identified in the Peruvian program would likely go undetected under Ecuador’s current passive surveillance approach. Based on this comparative evidence, we propose three concrete, sequenced steps for improvement: (1) as an immediate, low-cost measure, formalize symptom screening combined with GeneXpert testing at prison entry and exit points nationwide, prioritizing the highest-overcrowding facilities identified in Table 1 (Los Ríos and Guayas No. 5); (2) as a medium-term goal, pilot CXR-CAD-based biannual screening in the highest-burden facility (CPL Guayas No. 1), following the Peruvian protocol [19], given the demonstrated cost-effectiveness of this approach in comparable Latin American settings [20]; and (3), in parallel, integrate these diagnostic interventions with structural and decarceration policies, since modeling evidence indicates that biomedical interventions alone yield limited population-level impact where overcrowding remains structurally unaddressed [8].

Policy-level interventions may play a decisive role in reducing the tuberculosis burden associated with incarceration. Modeling studies suggest that decarceration policies could reduce future tuberculosis incidence at the population level by more than 10% in countries with prison systems similar to Ecuador [8]. These findings highlight the importance of integrating public health perspectives into criminal justice policies.

Ultimately, tuberculosis in Ecuadorian prisons represents not only a biomedical challenge but also a structural and social issue. Sustainable tuberculosis control will require coordinated action across health, justice, and social sectors to reduce overcrowding, strengthen prison healthcare systems, and ensure continuity of tuberculosis treatment during and after incarceration.

## 5. Conclusions

The evidence synthesized in this review makes one reality abundantly clear: correctional facilities act as powerful amplifiers of the tuberculosis epidemic, driven largely by structural neglect and delayed diagnoses. In Ecuador, local data paint a stark picture. Extreme overcrowding directly drives the exceptionally high incidence rates observed in major complexes like CPL Guayas No. 1.

However, a significant scientific blind spot remains. While the national circulation of drug-resistant strains is well documented, the true prevalence and transmission dynamics of multidrug-resistant tuberculosis inside these facilities remain uncertain due to a critical lack of prison-specific molecular surveillance.

Moving forward, the evidence demands a dual approach. Clinically, there is an urgent need to implement systematic active case finding using molecular tools to drastically reduce diagnostic delays. Ultimately, biomedical interventions alone cannot solve a structural crisis. These clinical measures will be insufficient unless they are integrated with public health and criminal justice policies aimed at definitively reducing overcrowding and improving prison healthcare infrastructure.

## Figures and Tables

**Figure 1:**
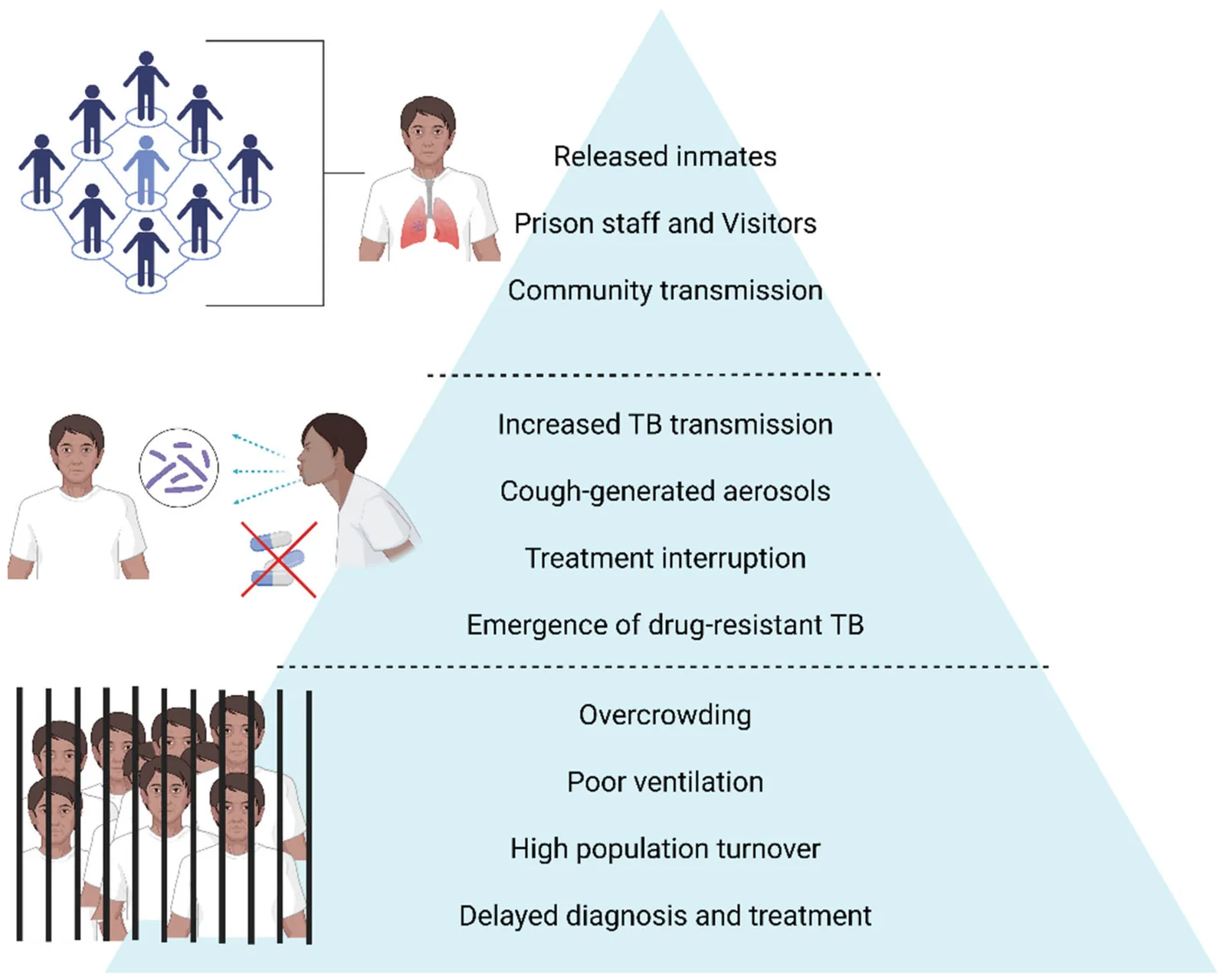
Conceptual pyramid illustrating tuberculosis amplification from prison environments to the community in Ecuador. Structural determinants within prisons, including overcrowding, poor ventilation, high population turnover, and delayed diagnosis and treatment, create conditions that facilitate sustained *Mycobacterium tuberculosis* transmission. These factors contribute to increased tuberculosis transmission, treatment interruption, and the emergence of drug-resistant strains within correctional facilities. At the population level, transmission extends beyond prison walls through released inmates, prison staff, and visitors, contributing to ongoing community transmission.

**Table 1:** Key structural and epidemiological indicators of tuberculosis risk in Ecuadorian prison settings.

Domain	Indicator	Value	Context	Source
Prison system capacity	Prison capacity	27,714 (2024); 27,138 (2025)	National prison system	[13]
Prison population	Total incarcerated population	32,941 (2024); 35,327 (2025)	Ecuador	
Overcrowding	National overcrowding rate	19% (2024); 30% (2025)		
Overcrowding (facility level)	Overcrowding rate	141%	Los Ríos prison	[14]
		124.6%	Guayas No.5 prison	
TB epidemiology	TB incidence	3947 per 100,000 inmates	CPL Guayas No.1	[7]
Treatment outcomes	Treatment success	65%		[7]
Treatment outcomes	Loss to follow-up	12.50%		[7]
Mortality	TB-related deaths	0.8%		
Health system delays	Median time from diagnosis → treatment	13 days (up to 2–3 months)		[7]

## Data Availability

No new data were created or analyzed in this study. Data sharing is not applicable to this article.

## Abbreviations

The following abbreviations are used in this manuscript:

TB	Tuberculosis
MTB	*Mycobacterium tuberculosis* complex
WHO	World Health Organization
HIV	Human immunodeficiency virus
AIDS	Acquired immunodeficiency syndrome
MDR	Multidrug resistant
XDR	Extensive drug resistant
PDL	People deprived of liberty
SNAI	National service for the comprehensive care of adults deprived of liberty
